# The Relationship of Self-Presentation, Psychological Needs, and Exercise Dependence in College Students With Overweight

**DOI:** 10.3389/fpsyg.2020.625501

**Published:** 2021-01-22

**Authors:** YiWen Liu, Hao Liu, ZhongQiang Liu

**Affiliations:** ^1^School of Physical Education, North Sichuan Medical College, Nangchong, China; ^2^School of Physical Education, Southwest Medical University, Luzhou, China

**Keywords:** overweight college students, self-presentation, psychological needs, exercise dependence, physical attractiveness

## Abstract

**Objectives:**

The objectives of the study were to explore the effect of self-presentation and psychological needs on exercise dependence and to provide an essential reference for preventing and inhibiting the production of exercise dependence in overweight college students.

**Methods:**

The freshmen in two comprehensive universities accepted physical fitness tests, filled out the scales of self-presentation, psychological needs, and exercise dependence after obtaining their consent. A sample of 747 overweight college students who have regular exercise was screened using the formula of Fox sports participation and the standard of overweight for Chinese adults. Multiple regression, exploratory factor, and confirmatory factor analyses were used to analyze the related data.

**Results:**

(1) In overweight college students, compared with the lower peers, those with higher physical attractiveness were more likely to suffer from detoxification of emotion, physiology, and vitality, and compared with the lower peers, those with higher self-presentation were more likely to appear in physiological abstinence. (2) The self-presentation of overweight male and female students has a significant positive influence on psychological needs (beta = 0.31, *p* < 0.01, and 0.37, *p* < 0.01, respectively, for males and females) and exercise dependence (beta = −0.21, *p* < 0.01, and 0.26, *p* < 0.01, respectively, for males and females). In contrast, psychological needs have a significant negative influence on exercise dependence (beta = −0.21, *p* < 0.01, and −0.26, *p* < 0.01, respectively, for males and females). (3) The psychological needs of overweight male and female college students were established as the mediating effect of self-presentation and exercise dependence. The mediating effect of psychological needs of females was higher than that of males (18.5 vs. 15.5%). The “ability display” of male and female students can affect “emotional distress” by “autonomy” and “competence.” The sense of relationship partially mediated the “attractiveness” of male and female students. Also, females on the one hand, rely on “weight control” by “competence” to produce some mediating effects on “physiological distress.” At the same time, the “ability display” by “competence” has a full mediating effect on “physiological distress.”

**Conclusion:**

In the self-presentation of the overweight college students, the higher scores in “attractiveness,” “weight control,” and “ability display,” the higher the psychological needs and exercise dependence; the higher the autonomy, competence, and relationship, the less the emotional, physiological, and dynamic distress.

## Introduction

Numerous studies have shown that the regular physical activities or fitness exercise are not only beneficial in reducing symptoms such as obesity, cardiovascular disease, cancer, and diabetes (Wang and Zhou, 2013; George and Kristi, 2017; Tang, 2017) but also in reducing anxiety, depression, and negative emotions, as well as enhancing self-esteem and cognitive function (Anthonieta et al., 2016; Chittrakul et al., 2020). However, although regular exercise can bring many benefits to people, excessive sports and physical activity will also induce psychological and physiological problems (Astrid et al., 2014; Badau and Badau, 2018). The symptom is termed as exercise dependence or addiction, which has been described as a positive addiction, but it can have links with damaging dysfunctional and excessive exercise behaviors (Hamer and Karageorghis, 2007; Astrid et al., 2014). Exercise dependence is a stereotyped unhealthy behavior pattern, usually manifested as an individual exercising excessively under an impulse that cannot be controlled (Li and Ma, 2011; Antunes et al., 2016). It is characterized by an inability to tolerate behavior interruption and withdrawal symptoms such as fatigue, irritability, insomnia, or loss of passion (Christopher et al., 2016; Blackstone and Herrmann, 2020). Reche-Garcia et al. (2018) pointed out that college students with obesity over-emphasized weight control or insist on exercising after injury, and the tolerance will be improved by means of compulsive thoughts; the tolerance will make adolescents with exercise dependence repeat a single form of exercise, and it is easy to lose control causing anxiety (Hamer and Karageorghis, 2007). In order to obtain a good body image in front of classmates or partners, overweight or obese adolescents often overemphasize body shape, appearance, and others’ evaluations. If exercise participation failed to achieve self-efficacy, body shape satisfaction, and relationship development needs, then their excessive exercise behavior may form dependent traits by compulsive impulse (Hamer and Karageorghis, 2007; Kristin and Tracy, 2014).

Self-presentation, also known as image management, is intended to form impressions from oneself to others, which has the process of eagerness to present a good impression to others and obtain the expected interactive results (Jay-Lee et al., 2012). Studies have found that in order to obtain a good impression of desire, individuals often use impression motivation to increase the amount of exercise and repeat the same exercise and other extreme exercise behaviors (Hausenblas and Giacobbi, 2004; Lodewyk, 2018). Self-presentation is an important mechanism of social psychology (Liu et al., 2015). Through image transmission and the acquisition of interactive results, people can adapt to development in the environment. Related literature pointed out that various populations use exercise to change their appearance and body shape (e.g., fat, thin) to enhance others’ impressions on them. The more special one is that they are affected by the stereotype of beauty. Adolescents and young women always use sports to form their body shapes to meet society’s current expectations and evaluations. Self-presentation is mainly composed of attractiveness (referring to showing oneself with body curves and muscle shapes), weight control (referring to presenting oneself with others’ perceptions of weight and evaluation), ability display (referring to exercise ability presenting themselves), and other three-dimensional structure (Niu et al., 2015; Liu et al., 2015). Previous studies have shown that in the process of self-presentation of college students, in order to increase the perception of body image and improve self-evaluation, those with low body shape satisfaction tended to induce body shape anxiety, which led to excessive exercise and stereotyped behavior. Once the exercise was blocked, exercise withdrawal will occur (Hausenblas and Fallon, 2006; Miller and Fry, 2018).

Self-determination theory advocates that motivation comes from psychological needs, namely, desire for self-potential growth and fulfilling internal needs will stimulate the engine to meet behavioral goals (Katie and Patrick, 2015; Magnus et al., 2016). Among them, sense of autonomy (referring to the degree of control behavior), need for competence (referring to the perception of the ability to interact with the environment), and sense of relationship (referring to the degree of desire to connect with others) are the main dimensional structures of psychological needs (Ryan and Deci, 2000; Vlachopoulos and Michailidou, 2006). These dimensions of self-presentation are in a positive and negative relationship with intrinsic and extrinsic motivations, respectively (Liu et al., 2015). Moreover, the dimensions of self-presentation affect sports participation through the degree of autonomy, that is, they induce intrinsic motivation through psychological needs and increase the frequency and time of sports participation. Some studies pointed out that relationship, competence, and autonomy are critical internal needs for establishing, nurturing, and maintaining regular exercise (Edmunds et al., 2008); these needs are for satisfying interest, having fun, and inducing behavior without compulsive thoughts and emotional impulses. It can reduce the risk of exercise-dependent symptoms (Niu et al., 2015).

As discussed above and observed in this study, it is not difficult to find that regular exercisers or people with fitness habits are high-risk groups of exercise dependence. The goal of most individuals in the exercise dependence group is to change their appearance and body shape (such as fat and thin) to improve body image and reduce body shape anxiety. Some scholars overseas attached great importance to the research on the causes and symptoms of exercise dependence (Hausenblas and Giacobbi, 2004; Hausenblas and Fallon, 2006; Badau and Badau, 2018). They gradually explored the relationship between self-presentation, psychological needs, and exercise dependence, and achieved some useful research results. At present, few scholars in China pay attention to this aspect. A few empirical studies (Li and Ma, 2011; Li et al., 2012) have measured the influencing factors of exercise dependence, and currently, few college counselors and clinical medical staff in school hospitals care about this matter. On the other hand, in the current research of domestic and overseas scholars, its research topics mainly focus on the relationship between self-presentation, exercise dependence disorder, and social body anxiety, the relationship between body image and exercise dependence, as well as the relationship between the three symptoms of the disease. In research topics on teenagers’ physical activities and exercise, most scholars aimed at participants in common fitness exercises, whereas for sex differences in overweight teenagers engaging in regular exercise, how does self-presentation affect their exercise dependence? Do psychological needs play a mediating role in the influence of self-presentation on exercise dependence? Those have little relevant reports in the literature.

University is a crucial period for the development of values. Sports are one of the life habits of college students. Many people do sports and physical activities intensely and even forget to eat (Liu et al., 2015; Niu et al., 2015). Many of these college students will construct a sense of self-identity from the results of many actions or behaviors to establish self-image and values. The sports and exercise provide a lot of information in this area. Some people will get achievements and affirmation from it, while others will get advice and criticism. However, sports have two sides. If you cannot properly plan, participate in an exercise in an appropriate amount at the right time, and participate healthily and safely, it can quickly become an abuse due to excessive dependence (Bane and McAuley, 1996). This study found that some college students are always affecting their academic performance or daily life due to excessive physical exercise. The reason for this symptom was that they craved for showing themselves through body shape, and even more so, avoiding the pressure of schoolwork, and even used the physical activity as an excuse for the decline in school performance. Based on the above discussion, this study will explore the following two issues: (1) explore the differences in the performance of overweight students of different genders and different levels of self-representation; (2) reveal the mediating effect of psychological needs on the effect of self-presentation to exercise dependence in overweight students. These explorations will provide some theoretical foundation and suggestions to the related governing body.

## Materials and Methods

### Participants

College students in the freshmen, sophomores, and juniors from two provincial universities in Sichuan, China participated in the present study. Sampling was carried out according to teaching classes from medical, science, arts, management, law, engineering, and education college, a total of eight colleges.

The survey procedures were divided into two steps:

The first step was the recruitment of the teachers in charge of the questionnaire survey. After consulting with the teachers in the public sports department, 14 teachers from two universities agreed to participate. All these teachers have been instructed on how to carry out this survey. When students have finished their routine fitness test, the physical education teachers gathered the students according to class, and then explained this survey procedure and purpose. Those who agreed to participate in this survey remained for the further survey. Each participant provided a written informed consent before the survey, then each participant filled out the questionnaires, and the teachers collected the questionnaires on the spot. This survey procedure took the participants about 25 min to finish.

The second step was the selection of overweight students based on whether they regularly exercised or not: (1) After the questionnaires were analyzed, the participants exercise participation level was determined using the Fox (1987) exercise participation level formula: exercise participation level = frequency × (time + intensity); the score must be ≥35 points. (2) Based on the Chinese adult overweight standards (Cheng-Ye and Jun-Ling, 2004), that is, BMI ≥ 25 (male) and BMI > 24 (female). In the end, this study recruited a sample of 747 students. Based on the scores of the three construct scale of “Physical Activity Self-Presentation Scale,” the first and last 27% of the participants were classified as high- and low-score groups: the individuals of males and females in the high and low groups in “physical attractiveness” were 174 vs. 179 and 119 vs. 133, respectively, 189 vs. 153 and 120 vs. 116, respectively in “weight control;” 221 vs. 207 and 145 vs. 126, respectively, in “ability display.”

### The Dimensional Structure and Validity of Scales

#### The Questionnaire for Sports Participation

The questionnaire for sports participation was developed by Fox (1987), which contains three items; the score range is between 2 and 72. The higher the score, the higher the degree of involvement. The test contents are exercise frequency, e.g., the times of exercises per week: “1” represents 0 times per week, “2” represents one time per week, “3” represents two times per week, “4” represents three times per week, “5” represents four times a week, “6” represents more than five times a week, a total of six options; exercise duration, e.g., the average time spent in each session: “1” represents 0–10 min per session, “2” represents 11–20 min per session, “3” represents 21–30 min per session, “4” represents 31–40 min per session, “5” represents 41–50 min per session, and “6” represents more than 51 min per session, a total of six options also; exercise intensity, e.g., the degree of fatigue after each exercise: “1” represents extremely relaxed, “2” represents very relaxed, “3” represents relaxed, “4” represents somewhat tired, “5” represents very tired, “6” represents extremely tired, a total of six options also. The retest reliability of the questionnaire was high, and its correlation coefficient *r* = 0.89.

#### The Self-Presentation Scale

The self-presentation scale was developed by Hu and Huang (2007). It contains three dimensions: physical attractiveness (eight items), e.g., “I have the confidence to show my body shape in front of others;” weight control (five items), e.g., “It is embarrassing to say my weight in front of others;” ability display (three items), e.g., “I feel that when I exercise in front of others, I will not be considered weak.” The scale uses a five-point Likert scale ranging from “1” (strongly disagree) to “5” (strongly agree). The higher the score, the higher the confidence level. The pre-test results showed that the scale could extract three common factors: the explanatory powers were 30.77, 22.87, and 17.5%, respectively. The cumulative explanatory power was 71.19%, and the three factors internal consistency Cronbach’s alpha coefficients were 0.88, 0.81, and 0.85, respectively. The overall Cronbach’s alpha coefficient was 0.79, and the overall test–retest reliability correlation coefficient *r* = 0.83. The confirmatory factor analysis of the measurement model showed that AGFI = 0.93, CFI = 0.95, NFI = 0.92, IFI = 0.94, and RMSEA = 0.039, indicating that the scale has good construct validity (see Table 1).

**TABLE 1:** Factor extraction and reliability analyses of three scales.

Scale	KMO and	Factors	Items	Eigenvalue	% of variance	Cumulative % of	Cronbach α
	Bartlett’s test				explained	variance explained	
Self-presentation	KMO = 0.87	Attractiveness	8	15.12	30.77	30.77	0.88
	*P* = 0.000	Weight control	5	11.24	22.87	53.64	0.81
		Ability display	3	8.62	17.55	71.19	0.85
Psychological needs	KMO = 0.82	Autonomy	6	12.06	39.87	39.87	0.83
	*P* = 0.000	Competence	5	8.25	21.75	61.62	0.80
		Relationship	5	5.05	13.32	74.94	0.86
Exercise dependence	KMO = 0.84	Emotional	3	11.44	28.57	28.57	0.86
	*P* = 0.000	Physiological	4	9.25	25.87	54.44	0.82
		Decrease vitality	5	6.09	17.03	71.47	0.83

#### The Psychological Needs for Exercise Scale

The three-dimensional scale developed by Vlachopoulos and Michailidou (2006) and modified by Yu (2013) was used. They are the sense of autonomy (six items), e.g., “The sports I engage in is that I like;” the sense of competency (four items), e.g., “I will be very effective in sports;” the sense of relationship (four items), e.g., “I feel very comfortable when I participate in sports with others.” The scale uses a five-point Likert scale ranging from 1 (strongly disagree) to 5 (strongly agree). The higher the score, the higher the perception. The pre-test results show that the scale could extract three common factors; the explanatory powers were 39.87, 21.75, and 13.32%, respectively. The cumulative explanatory power was 74.94%, and the three-dimensional internal consistency coefficient Cronbach’s alpha coefficients were 0.83, 0.80, and 0.86, respectively. The overall Cronbach’s alpha coefficient was 0.80, and the correlation coefficient of retest reliability was *r* = 0.86. A confirmatory analysis of the measurement model showed that AGFI = 0.95, CFI = 0.93, NFI = 0.94, IFI = 0.91, and RMSEA = 0.033, indicating that the scale has good construct validity (see Table 1).

#### The Exercise Dependence Scale

The scale developed by Li et al. (2012) was used to measure withdrawal symptoms within 24–36 h of being unable to exercise. The scale contains three dimensions, which are emotional distress (three items), e.g., “If I stop exercising for a few days, I will feel upset when I learn,” physiological distress (four items), e.g., “If I stop exercising for a few days, I will develop constipation,” and decreased vitality (five items), e.g., “If I stop exercising for a few days, my mobility will decrease.” The scale employed a five-point Likert scale to assess the exercise dependence: 1 = not at all; 2 = rarely; 3 = occasionally; 4 = often; 5 = always. The higher score indicated that the symptom would be more severe when unable to exercise. The pre-test results showed that the scale could extract three common factors. The explanatory powers were 28.57%, 25.87, and 17.03%, respectively. The cumulative explanatory power was 71.47%, and the three-dimensional internal consistency coefficients of Cronbach’s alpha were 0.86, 0.82, and 0.83, respectively. The overall Cronbach’s alpha coefficient was 0.84, and the correlation coefficient of retest reliability was *r* = 0.83. The confirmatory analysis of the measurement model showed that AGFI = 0.96, CFI = 0.95, NFI = 0.92, IFI = 0.93, and RMSEA = 0.031, indicating that the scale has good construct validity (see Table 1).

### Statistical Analyses

The data analyses in this study used SPSS Statistics 26.0 and AMOS 21.0 statistical analysis software. Continuous variables were reported as mean ± standard deviation. The normality tests have been carried using the Shapiro–Wilk test. To reveal the interaction effect of gender (female and male) and group (low and high score) of each construct in self-presentation on exercise dependence, the univariate two-factor analysis of variance was used; for the mixed gender, the difference in low and high score of each construct in self-presentation on exercise dependence was examined using independent sample *t*-test. Exploratory factor analysis (EFA), confirmatory factor analysis (CFA) factor, composite reliability (CR), and average variance extracted (AVE) were used to explore the path weights, direct and indirect effects of self-presentation, and psychological needs on exercise dependence, and the hierarchial regression analysis was used to reveal the mediating effect of psychological needs in the impact of self-presentation on exercise dependence. The classic mediating effect detection method was used to (1) examine whether self-presentation can significantly predict exercise dependence; (2) test whether self-presentation can significantly predict psychological needs; and (3) examine whether self-presentation and psychological needs can predict exercise dependence at the same time. The significance level of all indicator variables in this study was set to α = 0.05.

## Results

### The Differences of Exercise Dependence in Overweight Students by Gender and Self-Presentation Level

Table 2 shows that:

**TABLE 2:** Means scores and standard deviation of exercise dependence by low/high self-presentation and gender.

Gender	Self-presentation	Emotional distress	Physiological distress	Decreased vitality	Exercise dependence
Male	High attractiveness	8.54 ± 2.26	9.32 ± 3.21	14.54 ± 3.56	32.41 ± 7.25
	Low attractiveness	7.04 ± 2.33	8.41 ± 2.47	12.84 ± 3.54	28.29 ± 7.78
Female	High attractiveness	8.11 ± 2.58	9.37 ± 2.69	14.38 ± 3.58	31.86 ± 6.58
	Low attractiveness	7.43 ± 2.71	8.66 ± 2.82	13.27 ± 3.25	29.36 ± 6.32
Mixed	High attractiveness	8.28 ± 2.15*	9.11 ± 3.58*	14.34 ± 3.24*	31.73 ± 7.75*
	Low attractiveness	7.16 ± 2.18*	8.55 ± 2.27*	13.31 ± 3.69*	29.02 ± 6.14*
Male	High weight control	7.39 ± 2.24	8.22 ± 2.83*	13.33 ± 4.69	28.94 ± 8.69
	Low weight control	7.58 ± 2.69	8.38 ± 2.14	13.55 ± 3.75	29.51 ± 7.63
Female	High weight control	7.47 ± 2.69	8.91 ± 2.77*	13.98 ± 2.26	30.36 ± 5.96
	Low weight control	7.48 ± 2.58	8.55 ± 2.47	13.89 ± 3.63	29.92 ± 6.51
Mixed	High weight control	7.61 ± 2.25	8.50 ± 2.53	13.74 ± 3.64	29.85 ± 7.21
	Low weight control	7.53 ± 2.51	8.64 ± 2.17	13.61 ± 3.47	29.78 ± 6.06
Male	High ability display	7.81 ± 2.63	9.11 ± 2.14	13.47 ± 3.55	30.39 ± 7.25
	Low ability display	7.69 ± 2.17	8.33 ± 2.01	13.49 ± 3.08	29.51 ± 8.02
Female	High ability display	7.62 ± 2.58	8.79 ± 2.23	14.33 ± 3.47	30.74 ± 6.54
	Low ability display	7.59 ± 1.47	8.55 ± 2.78	13.79 ± 3.63	29.93 ± 6.02
Mixed	High ability display	7.74 ± 2.56	9.14 ± 2.47*	13.78 ± 3.37	30.66 ± 7.14
	Low ability display	7.62 ± 2.58	8.51 ± 2.47*	13.59 ± 3.38	29.72 ± 7.25

*p < 0.05.

(1) For the effects of physical attractiveness in self-presentation on exercise dependence, the gender effect on emotional distress, physiological distress, decreased vitality, and the overall exercise dependence did not reach a significant level. The four indicators’ *F*-values were 1.58, 0.77, 0.51, and 0.46, respectively. All corresponding significance levels were >0.05; the effect of high and low physical attractiveness on emotional distress, physiological distress, decreased vitality, and the overall exercise dependence reached a significant level. The *F*-values of the four indicators were 19.36 (*p* < 0.01), 12.25 (*p* < 0.01), 11.26 (*p* < 0.01), and 18.21 (*p* < 0.01), respectively. All the corresponding probability levels were less than 0.05. The interaction effect of gender × high/low physical attractiveness on emotional distress, physiological distress, decreased vitality, and the overall exercise dependence did not reach a significant level. The four indicators’ *F*-values were 0.97, 1.06, 0.87, and 0.66, respectively, and the corresponding probabilities were all *p* > 0.05. From the perspective of the mixed samples, those with high physical attractiveness score were significantly higher than those with low physical attractiveness in emotional distress, physiological distress, decreased vitality, and overall exercise dependence, which means that those with high physical attractiveness in the self-presentation of overweight students will have the higher symptoms of withdrawal in emotional distress, physiological distress, and decreased vitality.
(2) For the effects of weight control in self-presentation on exercise dependence, the gender effect on emotional distress, physiological distress, decreased vitality, and the overall exercise dependence did not reach a significant level. The four indicators’ *F*-values were 1.24, 0.68, 0.76, and 0.66, respectively. All Ps were greater than 0.05; the effect of high and low weight control on emotional distress, physiological distress, decreased vitality, and the overall exercise dependence reached a significant level. The four indicators’ *F*-values were 1.09, 12.77, 0.58, and 0.47, respectively. Only physiological distress reached a significant level. For the interaction effect of gender × high/low weight control on emotional distress, physiological distress, decreased vitality, and overall exercise dependence, the four indicators’ *F*-values were 0.78, 10.32, 0.65, and 0.74, respectively. Only physiological distress reached a significant level, and it showed that the high weight control on physiological distress in the females was higher than that in the males.
(3) For the effects of ability display in self-presentation on exercise dependence, the gender effect on emotional distress, physiological distress, decreased vitality, and the overall exercise dependence did not reach a significant level. The four indicators’ *F*-values were 0.83, 0.12, 1.14, and 0.29, respectively. The corresponding significant levels all were greater than 0.05; the effect of high and low ability display on emotional distress, decreased vitality, and the overall exercise dependence did not reach a significant level. The *F*-values of the three indicators were.0.32, 0.27, and 0.81, respectively, only physiological distress was 8.15 (*p* < 0.01); for the interaction effect of gender × high/low ability display on emotional distress, physiological distress, decreased vitality, and overall exercise dependence, the four indicators’ *F*-values were 0.85, 0.23, 0.21, and 0.44, respectively, their significance level were all greater than 0.05. From the perspective of the mixed samples, those with high ability display scores were significantly higher than those with low ability display in emotional distress, physiological distress, decreased vitality, and overall exercise dependence, which means that those with high ability display in the self-presentation of overweight students will have higher symptoms of withdrawal in emotional distress, physiological distress, and decreased vitality.

### The Impact of Self-Presentation and Psychological Needs on Exercise Dependence in Overweight College Students

#### Validation of the Overall Structure Model Diagram

Table 3 shows that:

**TABLE 3:** Statistical table of fit measures of male and female models.

Fit measures	Threshold	Male	Female
*X*^2^/*P*-value		1.85/*P* > 0.05	1.47/*P* > 0.05
GFI	≥0.9	0.94	0.95
AGFI	≥0.9	0.91	0.93
RMR	≤0.05	0.045	0.037
RMSEA	≤0.08	0.148	0.033
**Relative fit index**			
NNFI	≥0.9	0.93	0.95
CFI	≥0.9	0.94	0.97
**Parsimonious fit index**			
PNFI	≥0.5	0.583	0.514
PGFI	≥0.5	0.694	0.621
Hoelter CN	≥200	358.15	401.74

Fit measures of male and female’s self-presentation, psychological needs, and exercise dependence of the overall structure model demonstrate that: (1) In the absolute fit of the model test, the male and female chi-square values were 1.85 and 1.47, respectively, in turn, the significance level was less than 0.05, indicating that the overall model of male and female hypotheses was compatible with the actual data, besides, the absolute fit index GFI, AGFI, and SRMR values of male (female) were 0.94 (0.95), 0.91 (0.93), and 0.045 (0.037), respectively. They all reached the acceptable threshold. (2) In the relative fit of the model test, the male (female) NNFI and CFI were 0.93 (0.95) and 0.94 (0.97), respectively, which also reached the acceptable threshold. (3) In the parsimonious fit of the model test, the male (female) PNFI, PGFI, and CN values were 0.583 (0.514), 0.694 (0.621), and 358.15 (401.74), respectively, which all reached the acceptable threshold. The three tests mentioned above showed that the overall structural model adaptation of men and women was adequate.

Table 4 shows the fit measures of the internal structure of the male and female overall models: (1) The composite reliability of the latent variables of male and female self-presentation, psychological needs, and exercise dependence all exceeded 0.60, indicating that the internal quality of the model was relatively high. Thus, the model was ideal. (2) The average variance extracted shows that the self-presentation, psychological needs, and exercise dependence of the male were 0.48, 0.66, and 0.65, respectively, while those of the female were 0.56, 0.51, and 0.55, respectively. These show that the internal construct validity were relatively ideal.

**TABLE 4:** Statistical table of fit measures of the overall model test.

Scales	Factor	Regression weights		*R*^2^		1-*R*^2^		CR	AVE
						
		Male	Female	Male	Female	Male	Female	Male/female	Male/female
Self-presentation	Attractiveness	0.58	0.61	0.32	0.38	0.68	0.62	0.77/0.64	0.48/0.56
	Weight control	0.46	0.49	0.21	0.29	0.79	0.71		
	Ability control	0.69	0.70	0.47	0.49	0.53	0.51		
Physiological needs	Autonomy	0.75	0.68	0.56	0.48	0.44	0.52	0.80/0.76	0.66/0.51
	Competence	0.66	0.74	0.48	0.55	0.52	0.45		
	Relationship	0.84	0.66	0.74	0.49	0.36	0.51		
Exercise dependence	Emotional distress	0.78	0.64	0.66	0.48	0.34	0.52	0.87/0.79	0.65/0.55
	Physiological distress	0.75	0.76	0.62	0.57	0.38	0.43		
	Decreased vitality	0.73	0.64	0.60	0.47	0.40	0.53		

#### The Total Effect of Self-Presentation and Psychological Needs on Exercise Dependence

**FIGURE 1:**
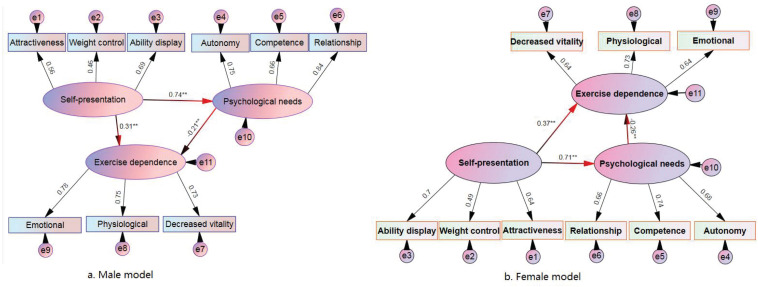
Standardized parameter estimates of regular exercise in overweight college students. **(A)** Male model, **(B)** female model.

Figure 1 shows that (1) for the overall path diagram of self-presentation and psychological needs on exercise dependence in the male, both self-presentation on psychological needs (standardized path coefficient β = 0.74, *p* < 0.01) and self-presentation on exercise dependence (β = 0.31, *p* < 0.01) reached significant levels, indicating that self-presentation affected psychological needs and exercise dependence, and the influence was positive, psychological needs and exercise dependence (β = −0.21, *p* < 0.05) also reached a significant level, indicating that psychological needs affected exercise dependence, and its effect was negative. (2) For the overall influence path of self-presentation and psychological needs on exercise dependence in the female, self-presentation on psychological needs (β = 0.71, *p* < 0.01) and self-presentation on exercise dependence (β = 0.37, *p* < 0.01) reached significant levels, indicating that self-presentation affected the psychological needs and exercise dependence, and the influence was positive; the psychological needs and exercise dependence (β = −0.26^∗^) also reached a significant level, indicating that the psychological needs affected the exercise dependence, and its influence was negative.

#### The Mediating Effect of Psychological Needs on Self-Presentation to Exercise Dependence

Table 5 shows that:

**TABLE 5:** Multiple regression analysis of self-presentation and physiological needs on exercise dependence.

		Self-presentation			Physiological needs			Statistical power		
		X_1_	X_2_	X_3_	Z_1_	Z_2_	Z_3_	*R*	*R*^2^	*F*
ED	Y_1_	0.25*/0.28*	0.04/0.08	0.34*/0.29*				0.54/0.52	0.29 / 0.27	8.78*/7.56*
	Y_2_	0.05/0.07	0.10/0.24*	0.30*/0.22*				0.49/0.60	0.24/0.36	7.58*/8.69*
	Y_3_	0.21*/0.27*	0.04/0.06	0.11/0.09				0.46/0.50	0.21/0.25	6.77*/7.32*
PN	Z_1_	0.19*/0.24*	0.06/0.12	0.21*/0.18*				0.54/0.56	0.29/0.32	6.09*/7.14*
	Z_2_	0.07/0.03	0.21*/0.26*	0.28*/0.20*				0.55/0.53	0.30/0.28	9.02*/6.69*
	Z_3_	0.16*/0.18*	0.23*/0.27*	0.31*/0.19*				0.54/0.58	0.29/0.34	11.03*/8.37*
ED	Y_1_	0.01/0.13	0.05/0.11	0.15*/0.16*	−0.25*/−0.26*	−0.22*/−0.19*	−0.05/−0.09	0.62/0.64	0.38/0.41	10.42*/15.21*
	Y_2_	0.24*/0.16*	0.01/0.18*	0.12/0.09	−0.01/−0.05	−0.20*/−0.26*	−0.14/−0.09	0.63/0.67	0.40/0.45	14.29*/15.21*
	Y_3_	0.15*/0.19*	0.05/0.08	0.08/0.13	−0.07/−0.11	−0.03/−0.06	−0.27*/−0.24*	0.69/0.65	0.47/0.42	13.95*/15.07*

ED, exercise dependence; PN, physiological needs. Y1: emotional distress, Y2: physiological distress, Y3: decreased vitality; X1: Physical attractiveness, X2: weight control, X3: ability display; Z1: autonomy, Z2: competence, Z3: relationship; “/” The data before and after represent male and female values, respectively. *P < 0.05.

(1) From the perspective of the direct impact of self-presentation on exercise dependence.

Male (female) regression equation 1 (Y_1_: emotional distress) was statistically significant: *F* = 8.78 (7.56), both *P* < 0.01. Among the three dimensions of male (female) self-presentation, attractiveness, and ability display reached a significant level. The standardized regression coefficients β were 0.25 (0.18), 0.04 (0.08), and 0.34 (0.29), respectively, and the determination coefficient *R*^2^ = 0.29 (0.27), indicating that attractiveness and ability display could explain male (female) emotional distress by 29% (27%) of the variance.

Male (female) regression equation 2 (Y_2_: physiological distress) was statistically significant: *F* = 7.58 (8.69), *P*< 0.01, in which the performance of the male and females’ self-presentation and weight control, and ability display of the females’ self-presentation were significant. The standardized regression coefficients β were 0.05 (0.07), 0.10 (0.24, *p* < 0.05), and 0.30 (0.22), respectively. The determination coefficient *R*^2^ = 0.24 (0.36), indicates that the male’s ability display could explain 24% of the variance of “physiological distress,” and the females’ weight control and ability display could explain 36% of their physical distress.

Male (female) regression equation 3 (Y_3_: decreased vitality) was very significant: *F* = 6.77 (7.32), *P* < 0.01; among the three dimensions of the male (female) self-presentation, only “physical attractiveness” reached the significance level. The standardized regression coefficients β were 0.21 (0.27), 0.04 (0.06), and 0.11 (0.09), respectively, and the determination coefficient *R*^2^ = 0.21 (0.25), indicating that physical attractiveness could explain 21% (25%) of the variation.

(2) From the perspective of the influence of self-presentation on psychological needs.

Male (female) psychological need regression equation 1 (Z_1_: autonomy) reached a significant level, [*F* = 6.09 (7.14), both *P* < 0.01], attractiveness and ability display in self-presentation reached a significant level, standardized regression coefficients β were 0.19 (0.24), 0.06 (0.12), and 0.21 (0.18), respectively, and the determination coefficient *R*^2^ = 0.29 (0.32), indicating that attractiveness and ability display could explain 29 and 32% of variation of the autonomy in male and female, respectively; male (female) regression equation 2 (Z_2_: competence) reached a significant level, *F* = 9.02 (6.69), *P* < 0.01, weight control and ability display in self-presentation reached a significant level, and standardized regression coefficients β were 0.07 (0.03), 0.21 (0.26), and 0.28 (0.20), respectively. The determination coefficient *R*^2^ = 0.30 (0.28), indicating that weight control and ability display could explain 30 and 28% of the variation in male and female competence; male (female) regression equation 3 (Z_3_: relationship) reached a significant level, *F* = 11.03 (8.37), *P* < 0.01, attractiveness, weight control, and ability display in self-presentation reached significant levels. The standardized regression coefficients β were 0.16 (0.18), 0.23 (0.27), and 0.31 (0.19), respectively. The determination coefficient *R*^2^ = 0.29 (0.34) indicates that attractiveness, weight control, and ability display could explain 29 and 34% of the variation of sense of relationship in males and females, respectively.

(3) The effect of both three dimensions of self-presentation (X_1_–X_3_) and three dimensions of psychological needs (Z_1_–Z_3_) on exercise dependence.

The regression equation Y_11_ (emotional distress) shows that “autonomy” and “competence” in the psychological needs of males and females have significant predictive power for “emotional distress.” At the same time, the “ability display” in self-presentation can significantly affect “autonomy” and “competence,” so it can be inferred that the “ability display” of males and females can affect emotional distress through “autonomy” and “competence” because the original direct regression equation Y_1_ normalized coefficient was 0.34^∗^ for males and 0.29^∗^ for females, which is still significant in equation Y_11_ but reduced to 0.15^∗^ and 0.16^∗^.

The regression equation Y_22_ (physiological distress) shows that the “competence” in the psychological needs of males (females) has significant predictive power on “physiological distress;” at the same time, the “weight control” and “ability display” of males and females significantly affect the “competence” since females’ “weight control” does not affect Y_2_. In contrast, females’ “weight control” and “ability display” have an impact on Y_2_. It can be inferred that males’ “ability display” entirely mediated “physiological distress” through “competence,” because males’ original direct regression equation Y_2_ has a standardized coefficient of 0.30, while in equation Y_22_, its standardized coefficient is −0.12, but it is no longer significant. Females’ “weight control” partially mediates “physiological distress” through “competence.” At the same time, females’ “ability display” completely mediates “physiological distress” through “competence” because the standardized coefficients of direct regression equation Y_2_ for females’ “weight control” and “ability display” were 0.24^∗^ and 0.22^∗^. In equation Y_22_, the coefficients are 0.18^∗^ (decreased but still significant) and −0.09 (decreased but no longer significant), respectively.

The regression equation Y_33_ (decreased vitality) shows that the “sense of relationship” in the psychological needs of males (females) has significant predictive power on the “decreased vitality.” At the same time, the “attractiveness” in self-presentation can significantly affect the “sense of relationship,” so it can be inferred that the “attractiveness” of males and females produces a partial mediating effect on “decreased vitality” through the “sense of relationship” because the standardized coefficients of theattractiveness of the direct regression for males (females) in equation Y_3_ were 0.21 (*p* < 0.05) and 0.27 (*p* < 0.05), respectively, while the coefficients in equation Y_33_ reduced to 0.15 (*p* < 0.05) and 0.19 (*p* < 0.05), but still significant.

## Analysis and Discussion

### From the Perspective of the Impact of Self-Presentation on Exercise Dependence by Gender

(1) Related research reported (Brewer et al., 2004; Molly et al., 2016) that exercise is a self-presentation strategy for contemporary college students. Its purpose is to create a healthy and lively image by changing body appearance and body shape through training to obtain a positive evaluation from peers and the opposite sex. The overall structure model obtained in this study (Figure 1) showed that self-presentation on exercise dependence had a significant level in overweight college students. The standardized path coefficients of males and females were 0.31 (*p* < 0.01) and 0.37 (*p* < 0.01), respectively, indicating that self-presentation directly and positively affected exercise dependence. The higher the self-presentation, the higher the exercise dependence would be. This result is similar to the findings of some scholars (Hausenblas and Giacobbi, 2004; Park, 2018). The purpose of self-presentation is to gain social interaction and use image management to enhance others’ impression of themselves. If this effort is not adequate, one will doubt self-presentation ability, which will cause social body anxiety, which will trigger physical, mental, and social withdrawal.
(2) Since the “self-presentation” in this study is composed of attractiveness, weight control, and ability display (e.g., X_1_, X_2_, and X_3_), “exercise dependence” is made up of emotional distress, physical distress, and decreased vitality (e.g., Y_1_, Y_2_, and Y_3_). To reveal the relationship between self-presentation and exercise dependence, the researchers divided the subjects into two groups with high and low self-confidence based on the scores of X_1_, X_2_, and X_3_. The results found that people with high physical attractiveness had significantly higher exercise withdrawal symptoms in three aspects of emotional distress, physical distress, and decreased vitality. Using Y_1_ as the dependent variable, X_1_, X_2_, and X_3_ were regarded as the independent variables and then multiple regression was performed (Table 5). It was found that attractiveness and ability display have higher explanatory power for emotional distress (Y_1_) (males and females for 29 and 27%, respectively), which indicated that if overweight male and female college students used physical attractiveness and ability display as the self-presentation strategy, once their exercise behavior was interrupted, they would have emotional distress. Astrid et al. (2014) pointed out that physical attractiveness is the use of body size and appearance to express oneself, while ability display is the use of athletic ability to present oneself. Both self-presentation strategies are significantly related to social body anxiety. More similar studies have shown (Gammage et al., 2004; Lauren et al., 2016; Alysse et al., 2017) that social body anxiety can generate emotional disorder symptoms, that is, when you perceive that others have a negative evaluation of your body’s appearance, they cannot recognize your abilities. It is easy to produce negative emotions such as sadness and depression due to increased anxiety. Therefore, the findings of this study further prove the findings of the previous scholars.
(3) The regression equation Y_2_ of this study showed that weight control and ability display (i.e., X_2_, X_3_) had a high explanatory power (36%) of physiological distress (Y_2_) in the overweight female college students. Ability display had significant explanatory power (24%) for overweight males’ physiological distress (notably, weight control did not affect males’ physical distress), this showed that if the overweight females use weight control and ability display, and the overweight males use the ability display as a self-presentation strategy, their exercise behavior will have physical stress once they are interrupted. Simultaneously, the data in Table 2 also clearly show that females have higher physical distress than males (*P* < 0.05) among the high weight control. Those with high ability display than low ability display also show as having higher physical pain (*P* < 0.05). Previous related studies also support these findings. Brewer et al. (2004) pointed out that the focus of female college students’ self-presentation is the evaluation of their body appearance and body curve. Gammage et al. (2004) found that females’ primary reason to participate in sports is to control their weight and show their charm through body shape. Alysse et al. (2017) found that when women are unable to successfully present an appearance that meets their own or social expectations, their social body anxiety questions the effect of the change, and then forces themselves to excessively increase the amount of exercise, while boys pay more attention to display, in the process of self-presentation of manliness, not being slim and thin. Lauren et al. (2016) pointed out that it is easy for college students to use athletic ability as a self-presentation strategy to attract peers’ attention by displaying superb skills, thereby enhancing self-image. Based on the above research results, it can be inferred that girls have stronger self-confidence in weight control and excessive exercise induced by their social body anxiety makes them more likely to have logical withdrawal symptoms than boys. Overweight female college students have higher self-confidence in weight control, and the higher their lives are troubled. Similarly, overweight male and female college students who have an increased ability to display self-presentation confidence are often more likely to have physical distress than low-ability presentations.

### From the Perspective of the Impact of Self-Presentation on Psychological Needs by Gender

Psychological needs are composed of three dimensions of autonomy, competence, and relationship. Autonomy refers to the individual’s perception of the degree of control over their behavior, that is, the individual is the initiator of the action and has the idea of free choice, and the actions are self-consistent. Competence refers to how an individual feels to manipulate and master the external environment, and whether the individual thinks their ability can effectively respond to exterior needs. The sense of relationship refers to whether an individual can emotionally be connected with others in behavior. When the environment provides enough acceptance, care, and warm emotional power, it can encourage the individual to accept various difficulties and challenges, and achieve mental growth.

The overall structure model diagram of this study (Figure 1) shows that the path of influence of male and female overweight students’ self-presentation on psychological needs has reached a significant level, with standardized path coefficients of 0.74 (*p* < 0.01) and 0.71 (*p* < 0.01), respectively, that is, self-presentation had a direct and positive influence on psychological needs. It manifested that the higher the self-presentation, the higher the psychological needs would be. The multiple regression equation shows the three dimensions of psychological needs (autonomy, competence, and relationship, i.e., Z_1_, Z_2_, and Z_3_) as the dependent variable, and the three dimensions of self-presentation (X_1_, X_2_, X_3_) as the independent variables (see Table 5): Attractiveness and ability display have a high positive explanatory power (29 and 32%) for overweight male and female college students’ autonomy (Z_1_); weight control and ability display have a higher positive effect on overweight male and female college students. Competence (Z_2_) has a high positive explanatory power (30, 28%); attractiveness, weight control, and ability display have a high positive explanatory power (29 and 34%) on the relationship between overweight male and female college students (Z_3_). Because the purpose of college students’ self-presentation is to show their ability, express themselves, and improve their self-worth (Bane and McAuley, 1996). According to the self-determination theory, this behavior goal has intrinsic demand and can induce inherent motivation to guide corresponding behavior. This behavior has a high autonomous control component (Deci and Ryan, 2000), so self-presentation and psychological needs have a specific connection. Besides, the sense of competence is the perception of the ability to interact with the environment. According to Edmunds et al. (2008), if the behavior goal can satisfy the competence, it will produce self-affirmation and a sense of accomplishment. In this way, even if the individual’s behavior is blocked, noother related symptoms will appear. In short, this study further confirmed that psychological needs would be elevated due to self-presentation. Suppose overweight college students engaging in regular exercise use physical exercise as a self-presentation strategy, their psychological needs would be higher. The higher the level of constructive self-presentation with attractiveness, weight control, and ability display, the higher the autonomy, competence, and sense of relationship.

### From the Perspective of the Effect of Psychological Needs on Exercise Dependence by Gender

(1) The overall structure model diagram (Figure 1) shows that the psychological needs of overweight male and female college students have a significant impact on exercise dependence, and the standardized path coefficients were −0.21 (*p* < 0.01) for male and −0.26 (*p* < 0.01) for female, that is, psychological needs will directly and negatively affect exercise dependence, which means that the higher the psychological demand, the lower the exercise dependence. According to a study by Hamer and Karageorghis (2007), psychological needs are the most primitive motivation to stimulate behavior. According to the self-determination theory, it will induce intrinsic motivation to guide behavior, so that the behavior will not be dominated by extrinsic motivation. Previous studies have confirmed that the higher the extrinsic motivation, the higher the tendency to exercise dependence, so psychological needs are closely related to exercise dependence. This study demonstrates that the causal relationship between psychological needs and exercise dependence indirectly confirms previous studies’ findings.
(2) To deeply reveal the influence of the three dimensions of psychological needs on the three dimensions of exercise dependence, this study again introduced multiple regression analysis (Y_11_, Y_12_, and Y_13_ in Table 5) and found that autonomy and competence have a significant adverse effect on emotional distress. The sense of competence has a significant negative explanatory power for physiological distress. In contrast, the sense of relationship has a significant negative explanatory power for decreased vitality. According to the theory of self-determination, motivation is derived from psychological needs. The higher autonomy, competence, and the sense of relationship will stimulate intrinsic motivation to cause behavior, and intrinsic motivation can inhibit the formation of exercise dependence (Brewer et al., 2004; Kyle et al., 2013; Lisa et al., 2014). For example, the sense of relationship is an inherent need to establish and develop a harmonious relationship, and the purpose of this need is to seek social support. When the perception is supported, the satisfaction of obtaining a balanced connection is higher. Therefore, once the behavior is blocked, it is less likely to produce withdrawal disorder. There will be no decline in vitality, so the sense of relationship can negatively predict the decreased vitality, explaining this study’s findings well. Therefore, from the results of this study, it is not difficult to infer that the higher the autonomy and competency needs in overweight students, the lower their emotional distress. The higher their competency needs, the lower their physical troubles. The higher their sense of relationship needs, the lower their vitality.

### From the Perspective of the Mediating Effect of Psychological Needs as Self-Presentation on Exercise Dependence

By using the classic three-step intermediary effect method, it is found (Table 5) that the “ability display” in self-presentation can partially influence the “emotional distress” in exercise dependence through the “autonomy” and “competence” in psychological needs in overweight students (regardless of male or female). The “ability display” in self-presentation of males can fully mediate the “physical distress” in “exercise dependence” through “competence” in “psychological needs.” In contrast, the “weight control” in “self-presentation” of overweight females has a partial mediation effect on the “physiological distress” in exercise dependence through the “competence” in psychological needs. Simultaneously, the “ability display” in the self-presentation of overweight females has a full intermediary on exercise dependence through the “competence” in psychological needs. The “physiological distress” of overweight students (regardless of male and female) can influence the “decreased vitality” in exercise dependence through the “sense of relationship” in psychological needs. Simultaneously, Figure 1 of the overall structure model further shows that the self-presentation of overweight (male and female) college students will indirectly and negatively affect exercise dependence through psychological needs. According to the standardized path coefficient, the mediating effect of male and female psychological needs were −0.155^∗^ and −0.185^∗∗^, respectively. The mediating impact of females’ psychological needs is higher than that of males, which, as a whole, proved that the self-presentation of overweight male and female college students has a strong inhibitory effect on exercise dependence through the intermediary effect of psychological needs. The intermediary effect of males and females can reach 15.5 and 18.5%, respectively.

### Limitations

(1) This research was aimed at a population of overweight college students, but the self-presentation process of

varying populations may be inconsistent. Are other populations the same as the results of this research, that is, is exercise dependence caused by self-presentation? It needs future research to explore further and verify it.
(2) In the previous self-presentation research category, there are also other aspects such as motivation and effectiveness. This research only explored three aspects. In the future, we can choose a more comprehensive scale to understand the effect of self-presentation on sports from more perspectives—the influence mechanism and development of exercise dependence.
(3) Owing to the limitations of the sample selection of the population, the overweight students in this study are just from two comprehensive universities, affecting the inference scope. Future studies should further expand the sample range to test the universality of this study’s results.

## Conclusion

(1) Self-presentation and psychological needs in overweight college students can positively and negatively affect exercise dependence. In the process of self-presentation, if the emphasis is placed on ability display and physical attractiveness, once the exercise is interrupted, there will be withdrawals such as emotional distress, physiological distress, or decreased vitality symptoms. If sports participation can meet the corresponding psychological needs of autonomy, competence, and relationship, it can inhibit the emotional, and physical distress caused by exercise interruption.
(2) There is a specific gender difference in self-presentation and psychological needs on exercise dependence in overweight college students. It is manifested that females with higher confidence in weight control and self-presentation have more physiological troubles than males; the psychological needs of overweight students are used as self-presentation for the effect of mediating exercise dependence, and reducing exercise dependence is established, but the mediating effect of the psychological needs of females is higher than that of boys.

### Implication and Suggestions

(1) In the process of self-presentation, overweight students do not overuse ability display and body appearance strategies. Otherwise, it will easily lead to body anxiety and excessive exercise dependence. On the contrary, if exercise participation can meet the goals of autonomy, competence, and relationship through autonomy, perception of control, and social relations to gain a sense of control and pleasure, and reducing compulsive movement thoughts and impulse, then this will reduce the frequency of withdrawal symptoms.
(2) Related governing body in charge of physical education, school hospitals, and health care in colleges and universities should produce corresponding posters or regularly organize lectures to advocate correct concepts, formulation principles, design methods, and participation essentials for weight control and exercise prescriptions to correctly guide contemporary college students’ self-presentation so that they can safely enjoy the challenges, fun, and achievements of exercise, and avoid the formation of stereotyped behaviors that may cause exercise dependence withdrawal.
(3) The overweight female college students habitually use weight control to manage their image and show their charm. In the process, they should have a positive evaluation of themselves, not to mind negative evaluation information too much, so as not to reduce the satisfaction of body image, and then suddenly increase the amount of exercise or excessive reliance on training, so that once the training is interrupted, there will be physiological distress.

## Data Availability Statement

The original contributions presented in the study are included in the article/supplementary material, further inquiries can be directed to the corresponding author/s.

## Ethics Statement

Ethical review and approval was not required for the study on human participants in accordance with the local legislation and institutional requirements. The patients/participants provided their written informed consent to participate in this study.

## Author Contributions

YWL wrote the first draft. All authors performed the research design and survey and revised the manuscript.

## Conflict of Interest

The authors declare that the research was conducted in the absence of any commercial or financial relationships that could be construed as a potential conflict of interest.

## References

[B1] Alysse B. K. Kimberley L. G. Cathy V. I. (2017). How do you define body image? Exploring conceptual gaps in understandings of body image at an exercise facility. *Body Image* 23 69–79. 10.1016/j.bodyim.2017.08.003 28886392

[B2] Anthonieta L. M. Felipe N. C. Fíviade A. L. (2016). Investment in beauty, exercise, and self-esteem: are they related to self-perception as a romantic partner? *Evol. Psychol. Sci.* 2 24–31. 10.1007/s40806-015-0032-6

[B3] Antunes H. K. Leite G. S. Lee K. S. Barreto A. T. Santos R. V. Souza Hde S. (2016). Exercise deprivation increases negative mood in exercise-addicted subjects and modifies their biochemical markers. *Physiol. Behav.* 156 182–190. 10.1016/j.physbeh.2016.01.028 26812592

[B4] Astrid M. Brian C. Heike Z. Herberg A. Müller V. de Zwaan M. (2014). Does the German version of the exercise dependence scale measure exercise dependence? *Psychol. Sport Exerc.* 15 288–292. 10.1016/j.psychsport.2013.12.003

[B5] Badau D. Badau A. (2018). Identifying the incidence of exercise dependence attitudes, levels of body perception, and preferences for use of fitness technology monitoring. *Int. J. Environ. Res. Public Health* 15:2614. 10.3390/ijerph15122614 30469525 PMC6313704

[B6] Bane S. M. McAuley E. (1996). The role of efficacy cognitions in reducing physique anxiety in college female. *Med. Sci. Sport Exerc.* 28 85–83. 10.1097/00005768-199605001-00508

[B7] Blackstone S. R. Herrmann L. K. (2020). Fitness wearables and exercise dependence in college women: considerations for university health education specialists. *Am. J. Health Educ.* 51 225–233. 10.1080/19325037.2020.1767004

[B8] Brewer B. W. Diehl N. S. Cornelius A. E. Joshua M. D. Van Raalte J. L. S. Cornelius A. E. (2004). Exercising caution: social physique anxiety and protective self-presentational behavior. *J. Sci. Med. Sport* 7 47–55. 10.1016/S1440-2440(04)80043-4 15139164

[B9] Cheng-Ye J. I. Jun-Ling S. (2004). Dynamic analysis on the prevalence of obesity and overweight school–age children and adolescents in recent 15 years in china. *Chinese J. Epidemiol.* 25 103–108. 15132859

[B10] Chittrakul J. Siviroj P. Sungkarat S. (2020). Multi-system physical exercise intervention for fall prevention and quality of life in pre-frail older adults: a randomized controlled trial. *Int. J. Environ. Res. Public Health* 17 185–195. 10.3390/ijerph17093102 32365613 PMC7246743

[B11] Christopher A. M. Ian B. Lance B. (2016). Profiles of exercise dependence symptoms in Ironman participants. *Psychol. Sport Exerc.* 24 48–55. 10.1016/j.psychsport.2016.01.005

[B12] Deci E. L. Ryan R. M. (2000). The “what” and “why” of goal pursuits: human needs and self-determination of behavior. *Psychol. Inq.* 11 227–268. 10.1207/S15327965PLI1104_01

[B13] Edmunds J. Ntoumains N. Duda J. L. (2008). Testing a self- determination theory-based teaching style intervention in the exercise domain. *Psychol. Sport Exerc.* 30 101–112. 10.1002/ejsp.463

[B14] Fox K. R. (1987). *Physical Self-perceptions and Exercise Involvement.* Tempe: Arizona State University.

[B15] Gammage K. L. Hall C. R. Ginis K. A. M. (2004). Self-presentation in exercise contexts: differences between high and low frequency exercisers. *J. Appl. Soc. Psychol.* 34 1638–1651. 10.1111/j.1559-1816.2004.tb02791.x

[B16] George A. K. Kristi S. K. (2017). Exercise and cancer-related fatigue in adults: a systematic review of previous systematic reviews with meta-analyses. *BMC Cancer* 693 2–17. 10.1186/s12885-017-3687-5 29058631 PMC5651567

[B17] Hamer M. Karageorghis C. I. (2007). Psychobiological mechanisms of exercise dependence. *Sports Med.* 37 477–484. 10.2165/00007256-200737060-00002 17503874

[B18] Hausenblas H. A. Fallon E. A. (2006). Exercise and body image: a meta-analysis. *Psychol. Health* 21 33–47. 10.1080/14768320500105270

[B19] Hausenblas H. A. Giacobbi P. R. (2004). Relationship between exercise dependence symptoms and personality. *Pers. Indiv. Differ.* 36 1265–1273. 10.1016/S0191-8869(03)00214-9

[B20] Hu K. Y. Huang S. F. (2007). Analysis of reliability and validity of physical activity self presentation self-confidence scale. *Natl. Inst. Phys. Educ. Forum* 16 303–331.

[B21] Jay-Lee L. Robert G. J. James A. D. (2012). Trait perfectionism, self-determination, and self-presentation processes in relation to exercise behavior. *Psychol. Sport Exerc.* 13 224–235. 10.1016/j.psychsport.2011.11.003

[B22] Katie E. G. Patrick G. (2015). Testing a bifactor model to disentangle general and specific factors of motivation in self-determination theory. *Pers. Indiv. Differ.* 81 35–40.

[B23] Kristin J. H. Tracy L. T. (2014). Appearance-based exercise motivation moderates the relationship between exercise frequency and positive body image. *Body Image* 11 101–108. 10.1016/j.paid.2014.12.059 24529336

[B24] Kyle F. P. Lisa M. C. Luc J. M. Hall C. R. (2013). Too much of a good thing? Examining the relationship between passion for exercise and exercise dependence. *Psychol. Sport Exerc.* 14 493–500. 10.1016/j.psychsport.2013.02.003

[B25] Lauren N. F. April R. S. Lauren M. F. Elhai J. D. Rhudy J. L. Teng E. T. (2016). Using implicit attitudes of exercise importance to predict explicit exercise dependence symptoms and exercise behaviors. *Psychol. Sport Exerc.* 22 91–97. 10.1016/j.psychsport.2015.06.006 26195916 PMC4505176

[B26] Li M. L. Ma W. P. (2011). Analysis of influencing factors of regular exercise dependence of college students. *J. Tianjin Inst. Phys. Educ.* 26 546–548.

[B27] Li M. L. Ma W. P. Deng L. P. (2012). Development and reliability and validity analysis of exercise dependence scale. *J. Tianjin Inst. Phys. Educ.* 27 360–365.

[B28] Lisa M. Celine M. B. Amanda B. (2014). Do portrayals of women in action convey another ideal that women with little self-determination feel obligated to live up to? Viewing effects on body image evaluations and eating behaviors. *Appetite* 83 277–286. 10.1016/j.appet.2014.08.025 25178899

[B29] Liu Q. Q. Sun X. J. Zhou Z. K. Niu G. F. (2015). The effect of self presentation in social networking sites on adolescents’ self-identity: the role of online positive feedback. *Chinese J. Clin. Psychol.* 23 1094–1097. 10.16128/j.cnki.1005-3611.2015.06.032

[B30] Lodewyk K. R. (2018). Associations between trait personality, anxiety, self-efficacy and intentions to exercise by gender in high school physical education. *Educ. Psychol.* 38 487–501. 10.1080/01443410.2017.1375081

[B31] Magnus L. Karin W. J. Simon J. S. Martyn S. (2016). Viewing exercise goal content through a person-oriented lens: a self-determination perspective. *Psychol. Sport Exerc.* 27 85–92. 10.1016/j.psychsport.2016.06.011

[B32] Miller S. Fry M. (2018). Relationship between motivational climate to body esteem and social physique anxiety within college physical activity classes. *J. Clin. Sport Psychol.* 12 525–543.

[B33] Molly V. Driediger, Carly D. Mckay, Craig R. H. Echlin P. S. (2016). A qualitative examination of women’s self-presentation and social physique anxiety during injury rehabilitation. *Physiotherapy* 102 371–376. 10.1016/j.physio.2015.10.001 26608591

[B34] Niu G. F. Bao N. Fan C. Y. Zhou Z. K. Kong F. C. Sun X. J. (2015). The effect of self presentation on self-esteem in social networking sites: the mediating role of social support. *Psychol. Sci.* 38 939–945. 10.16719/j.cnki.1671-6981.2015.04.025

[B35] Park J. (2018). Emotional reactions to the 3D virtual body and future willingness: the effects of self-esteem and social physique anxiety. *Virt. Real.* 22 1–11. 10.1007/s10055-017-0314-3

[B36] Reche-Garcia C. Ortin Montero F. J. Martinez-Rodriguez A. (2018). Aspects related with physical exercise dependence in university students. *Sport Rev. Euroam. Cienclas Dep.* 7 39–44.

[B37] Ryan R. M. Deci E. L. (2000). Self-determination theory and the facilitation of intrinsic motivation, social development, and well-being. *Am. Psychol.* 55 68–78. 10.1037/0003-066X.55.1.68 11392867

[B38] Tang J. (2017). Relationship between polymorphism of AAS gene and antihypertensive effect of aerobic exercise with different intensity in male patients with essential hypertension. *J. Beijing Sport Univ.* 40 46–57.

[B39] Vlachopoulos S. P. Michailidou S. (2006). Development and initial validation of a measure of autonomy, competence, and relatedness in exercise: the basic psychological needs in exercise scale. *Meas. Phys. Educ. Exerc. Sci.* 10 179–201. 10.1207/s15327841mpee1003.4

[B40] Wang Z. Z. Zhou Y. (2013). Exercise, physical activity and prevention of chronic diseases. *J. Wuhan Inst. Phys. Educ.* 47 69–75. 10.16930/j.cnki.wtxb.2013.11.001

[B41] Yu C. Y. (2013). Preliminary revision on elderly fitness mental demand satisfaction scale. *China Sport Sci.* 33 88–96. 10.3969/j.issn.1000-677X.2013.07.013

